# Elevational Metabolic Reprogramming Optimizes Flavonoid Accumulation and Antioxidant Capacity in *Chimonobambusa utilis* Leaves

**DOI:** 10.3390/plants15091290

**Published:** 2026-04-22

**Authors:** Jingkai Wu, Yingying Dai, Boqian Qin, Songming Li, Jianjun Zhang, Fanfan He, Guohua Liu, Changlai Liu

**Affiliations:** 1 National Key Laboratory for the Development and Utilization of Forest Food Resources, Co-Innovation Centre for Sustainable Forestry in Southern China, Bamboo Research Institute, Nanjing Forestry University, Nanjing 210037, China; jkw610@njfu.edu.cn (J.W.); daiyy@njfu.edu.cn (Y.D.); w1091519521@163.com (B.Q.); 3211700723@njfu.edu.cn (J.Z.); 8231711769hff@njfu.edu.cn (F.H.); ghliu@njfu.edu.cn (G.L.); 2 Yibin Forestry and Bamboo Industry Research Institute, Yibin 644005, China

**Keywords:** *Chimonobambusa utilis*, bamboo leaves, altitudinal gradient, widely targeted metabolomics, flavonoids, redox homeostasis

## Abstract

*Chimonobambusa utilis* is a dominant bamboo species in China, yet its leaves remain an underutilized resource despite their significant bioactive potential. To elucidate the metabolic reprogramming of *Ch. utilis* leaves across an elevational gradient and its link to antioxidant phenotypes, we integrated widely targeted metabolomics with redox profiling of leaves collected from 1150, 1600, and 2000 m in the Qingba Mountains. The mid-elevation (1600 m) group exhibited the most robust antioxidant capacity and the highest total flavonoid content. Metabolomic analysis identified 3113 metabolites across 13 classes, with flavonoids (604 compounds, 22.7% of total abundance) emerging as the predominant secondary metabolites. Pairwise comparisons revealed 1716 differentially accumulated metabolites (DAMs). KEGG enrichment indicated that while the low-elevation (1150 m) group prioritized primary metabolism and upstream phenylpropanoid branches, the high-elevation (2000 m) group was associated with photoprotection and defense responses. In contrast, the mid-elevation environment optimized the flux toward flavonoid biosynthesis while maintaining steady metabolic supply. HPLC quantification further confirmed that key markers—vitexin, hyperoside, orientin, and luteoloside—peaked at 1600 m. Correlation analysis between 423 differential flavonoids and antioxidant indices demonstrated that distinct radical-scavenging activities are driven by specific flavonoid structural motifs. Overall, altitude-driven metabolic remodeling, characterized by a mid-elevation advantage for flavonoid accumulation, dictates the antioxidant plasticity of *Ch. utilis* leaves.

## 1. Introduction

*Chimonobambusa utilis* (Poaceae: Bambusoideae: *Chimonobambusa*), popularly known as the “King of Bamboo Shoots,” is one of the most economically significant bamboo species in southwestern China, covering an expansive plantation area of approximately $6.67\times 10^{4}$ ha. While its shoots are highly prized for their unique flavor and rich trace elements, the extensive leaf biomass—often discarded during timber and shoot harvesting—represents a vastly underutilized bioresource. This species forms extensive pure stands across a vertical gradient of 1100–2100 m [1], making it not only a backbone of the regional bamboo industry but also an ideal natural system for examining how altitudinal gradients modulate plant physiological and metabolic processes. As altitude increases, irradiance and UV exposure intensify, air temperature declines, and diurnal temperature variation becomes more pronounced. These coordinated environmental changes may disrupt the balance between reactive oxygen species (ROS) generation and scavenging in leaves, thereby perturbing cellular redox homeostasis [2,3]. In response, the reorganization of secondary metabolism constitutes a critical acclimatory strategy. In particular, the phenylpropanoid–flavonoid metabolic network is considered a central chemical hub underlying leaf photoprotection and redox regulation, closely linking external stress signals with physiological performance [4,5].

Bamboo leaves are rich in polyphenolic bioactives, particularly flavonoids and phenolic acids, which exhibit potent antioxidant properties [6,7]. In bamboos and related taxa, environmental gradients frequently drive the remodeling of polyphenol chemotypes. For example, in *Indocalamus latifolius*, altitudinal variation markedly affects the accumulation of chlorogenic acid and vitexin, shifting the leaf antioxidant phenotypes. Consistently, LC-QTOF-MS-based metabolomics has shown that altitude reshapes flavonoid distribution patterns across diverse bamboo species [8,9]. Structurally, bamboo leaf flavonoids share a common C_6_–C_3_–C_6_ backbone, and their antioxidant and radical-scavenging capacities are largely determined by B-ring hydroxylation patterns and the conjugated system formed by the C2 = C3 double bond and the 4-oxo group in the C-ring [10]. Importantly, bamboo leaf extract (BLE) has been included in the Chinese national standard (GB 30615-2014 [11]) as a food-grade antioxidant ingredient [12], highlighting the recognized antioxidant value and application potential of bamboo leaf flavonoids. Although altitudinal variation in phenolics and antioxidant traits has been reported in some bamboo and montane plant species, most studies have focused on total contents or a few targeted compounds rather than metabolome-wide changes. For *Ch. utilis*, evidence remains scarce regarding how elevational gradients reshape leaf metabolome composition, flavonoid accumulation, and antioxidant capacity in an integrated manner.

Metabolomics enables the comprehensive characterization of dynamic metabolic responses of plants to endogenous and environmental stimuli [13]. Among current analytical approaches, LC–MS/MS-based widely targeted metabolomics provides a useful balance between metabolite coverage, analytical throughput, and quantitative robustness, making it well suited for dissecting metabolic-network remodeling along environmental gradients [14]. In this study, we integrated leaf physicochemical traits, extract-based antioxidant assays (DPPH, ABTS, and reducing power), and widely targeted metabolomics to systematically investigate *Ch. utilis* leaves across an altitudinal gradient. Based on the hypothesis that altitudinal variation shapes redox capacity by driving specific flavonoid accumulation, this study aims to address: (1) how altitudinal gradients drive the overarching remodeling of the leaf metabolome; (2) the specific shifts within flavonoid biosynthetic pathways across elevations; and (3) how these variations and their structural features dictate distinct antioxidant capacities, linking metabolic reprogramming to the redox phenotype.

## 2. Materials and Methods

### 2.1. Plant Materials

Plant materials were collected on 18 December 2024 from Qingba Mountain, Tongzi County, Guizhou, China. Sampling was conducted on the same day to ensure that leaves from all altitudinal populations were collected under the same seasonal background while capturing a clear thermal gradient across elevations. On the sampling day, the air temperatures were approximately −6.2 °C at 2000 m, −1.8 °C at 1600 m, and 3.0 °C at 1150 m, and snow/ice were observed at the high-altitude site. Three sampling sites were established along an altitudinal gradient: high altitude (2000 m; 28°51′58.5946″ N, 107°03′46.5589″ E), mid altitude (1600 m; 28°50′41.0454″ N, 107°04′12.3805″ E), and low altitude (1150 m; 28°48′35.7621″ N, 107°05′25.2571″ E), hereafter referred to as GHB, ZHB, and DHB, respectively. At each altitude, three independent biological replicates were established. For each replicate, leaves were collected from three randomly selected *Chimonobambusa utilis* individuals with comparable growth status within the sampling site. From each individual, 300 leaves were collected, including 100 leaves each from the upper, middle, and lower canopy positions. Representative photographs of the sampled *Ch. utilis* plants and leaves are provided in Figure S3. The leaves from the three individuals were then pooled to form one composite replicate sample. Leaves were pooled only within each biological replicate, and no pooling was performed across the three biological replicates or across altitude groups. Each biological replicate was then divided into two portions. One portion of fresh leaves was rinsed with distilled water to remove surface dust and impurities, immediately frozen in liquid nitrogen, and stored at −80 °C for metabolomic analysis. The other portion was oven-dried at 50 °C, ground into powder, and passed through a 30-mesh sieve for the determination of total flavonoid and total phenolic contents. Unless otherwise stated, all metabolomic and biochemical analyses were performed using three independent biological replicates for each altitude.

### 2.2. Extraction of Total Flavonoids and Total Phenolics

Leaf powder (0.25 g) was placed in a 50 mL centrifuge tube and mixed with 26 mL of 90% (*v*/*v*) ethanol. After soaking in the dark at room temperature for 12 h, the mixture was subjected to ultrasound-assisted extraction for 69 min. The extract was then centrifuged at 7000 rpm for 15 min, and the supernatant was collected. The residue was re-extracted with 6.5 mL of 90% (*v*/*v*) ethanol under the same extraction and centrifugation conditions. The supernatants from the two extractions were combined (32.5 mL in total) to obtain the bamboo leaf extract, which was used for the determination of total flavonoids, total phenolics, and antioxidant activities, including DPPH radical-scavenging activity, ABTS radical-scavenging activity, and reducing power.

### 2.3. Determination of Total Flavonoid Content

Total flavonoid content (TFC) was determined using an aluminum chloride colorimetric method with minor modifications to [15]. Briefly, 3.0 mL of bamboo leaf extract was mixed with 0.3 mL of 10% (*w*/*v*) AlCl_3_ solution and diluted to 10.0 mL with 90% (*v*/*v*) ethanol. The mixture was incubated in the dark at 25 °C for 15 min. The blank contained 9.7 mL of 90% (*v*/*v*) ethanol and 0.3 mL of 10% (*w*/*v*) AlCl_3_. Absorbance was measured at 420 nm using a UV–Vis spectrophotometer (Shimadzu UV-1900i, Shimadzu, Kyoto, Japan) and recorded as $A_{TFC}$. A calibration curve was prepared using hyperoside as the standard (*y* = 37.66*x* + 0.1664, R^2^ = 0.997), TFC was expressed as mg hyperoside equivalents per g dry weight (mg HE/g DW) and calculated as follows:

$$C_{TFC}(mg\;HE/g)=\left(\frac{A_{TFC}-0.1664}{37.66}\right)\times 433.33$$

### 2.4. Determination of Total Phenolic Content

Total phenolic content (TPC) was determined using the Folin–Ciocalteu method with minor modifications to [15]. Briefly, 1.0 mL of bamboo leaf extract was mixed with 1.0 mL of Folin–Ciocalteu reagent and 2.0 mL of 20% (*w*/*v*) Na_2_CO_3_ solution. The mixture was reacted in a thermostatic water bath at 50 ± 1 °C for 30 min, then cooled and brought to 25.0 mL with distilled water. After mixing, the solution was incubated in the dark at 25 °C for 30 min. Absorbance was measured at 745 nm using a UV–Vis spectrophotometer and recorded as $A_{TPC}$. A calibration curve was prepared using gallic acid as the standard (*y* = 107.573*x* + 0.00817, R^2^ = 0.999), TPC was expressed as mg gallic acid equivalents per g dry weight (mg GAE/g DW) and calculated as follows:

$$C_{TPC}(mg\;GAE/g)=\left(\frac{A_{TPC}-0.00817}{107.573}\right)\times 3250$$

### 2.5. Redox Assays of Bamboo Leaf Extracts

#### 2.5.1. DPPH Radical-Scavenging Activity

DPPH radical-scavenging activity was measured with minor modifications to [16]. The sample group consisted of 1.0 mL of bamboo leaf extract mixed with 3.0 mL of DPPH solution and incubated in the dark at room temperature for 30 min; the absorbance at 517 nm was measured using a UV–Vis spectrophotometer and recorded as $A_{mix}$. In parallel, 1.0 mL of bamboo leaf extract was mixed with 3.0 mL of anhydrous ethanol, incubated in the dark under the same conditions, and the absorbance at 517 nm was recorded as $A_{BLE}$. The blank was prepared by mixing 1.0 mL of 90% (*v*/*v*) ethanol with 3.0 mL of DPPH ethanolic solution, followed by incubation in the dark under the same conditions, and the absorbance at 517 nm was recorded as $A_{blank}$. The DPPH radical scavenging activity was calculated as follows:

$$P_{DPPH}\left(\%\right)=\left(1-\frac{A_{mix}-A_{BLE}}{A_{blank}}\right)\times 100\%$$

#### 2.5.2. ABTS Radical-Scavenging Activity

ABTS radical-scavenging activity was measured with minor modifications to [7]. The sample group consisted of 0.4 mL of bamboo leaf extract mixed with 3.6 mL of ABTS solution and allowed to react in the dark at room temperature for 5 min; the absorbance at 734 nm was measured using a UV–Vis spectrophotometer and recorded as $A_{mix}$. The blank was prepared by mixing 0.4 mL of 90% (*v*/*v*) ethanol with 3.6 mL of ABTS solution, followed by incubation in the dark under the same conditions, and the absorbance at 734 nm was recorded as $A_{blank}$. The ABTS radical scavenging activity was calculated as follows:

$$P_{ABTS}\left(\%\right)=\frac{A_{blank}-A_{mix}}{A_{blank}}\times 100\%$$

#### 2.5.3. Reducing Power

Total reducing power was determined with minor modifications to [17]. Briefly, 1.0 mL of bamboo leaf extract was mixed with 1.0 mL of 0.2 mol L^−1^ phosphate buffer (pH 6.6) and 1.0 mL of 1% (*w*/*v*) potassium ferricyanide solution. After thorough mixing, the reaction was carried out in a thermostatic water bath at 50 ± 1 °C for 20 min. The mixture was then rapidly cooled, and 1.0 mL of 10% (*w*/*v*) trichloroacetic acid was added. The solution was centrifuged at 4800 r/min for 5 min, and 2.0 mL of the supernatant was transferred to a 5 mL centrifuge tube, followed by the addition of 0.4 mL of 1% (*w*/*v*) FeCl_3_ solution and 2.0 mL of distilled water. After standing for 10 min, the absorbance at 700 nm was measured and recorded as $A_{mix}$. For the control, distilled water was used to replace the potassium ferricyanide solution, and the absorbance was measured under the same conditions and recorded as $A_{blank}$. Total reducing power was expressed as the absorbance difference ($A_{700}$) and calculated as follows:

$$A_{700}=A_{mix}-A_{blank}$$

### 2.6. Determination of Soluble Sugar Content

Soluble sugar content (SSC) was determined using the anthrone–sulfuric acid method with minor modifications. Briefly, 0.1 g of dried leaf powder was placed in a 50 mL centrifuge tube and mixed with 15 mL of distilled water. After extraction in a boiling water bath for 20 min, the extract was filtered into a 100 mL volumetric flask. The centrifuge tube and residue were repeatedly rinsed with distilled water, and the combined extract was brought to volume with distilled water. An aliquot of 0.5 mL extract was transferred to a 20 mL stoppered test tube, mixed with 1.5 mL of distilled water, followed by the sequential addition of 0.5 mL anthrone reagent in ethyl acetate and 5.0 mL concentrated sulfuric acid. After thorough mixing, the tube was immediately placed in a boiling water bath for 10 min, then removed and cooled naturally to room temperature. Absorbance was measured at 620 nm using a UV–Vis spectrophotometer and recorded as $A_{SSC}$. A calibration curve was prepared using sucrose as the standard (*y* = 5.9867*x* + 0.0002, R^2^ = 0.998), SSC was expressed as mg sucrose equivalents per g dry weight (mg SE/g DW) and calculated as follows:

$$C_{SSC}(mg\;SE/g)=\left(\frac{A_{SSC}-0.0002}{5.9867}\right)\times 150$$

### 2.7. Metabolomics Sample Preparation

The frozen leaf samples used for metabolomic analysis were freeze-dried under vacuum for 63 h (Scientz-100F, Scientz, Ningbo, China) and then ground into fine powder (MM 400, Retsch, Haan, Germany; 30 Hz, 1.5 min). Powder (30 mg) was accurately weighed using an analytical balance (MS105DM, Sartorius, Göttingen, Germany) and extracted with 1500 µL of prechilled (−20 °C) 70% (*v*/*v*) methanol containing internal standards. The mixture was vortexed for 30 s every 30 min (six times in total) and then centrifuged at 12,000 rpm for 3 min. The supernatant was filtered through a 0.22 µm membrane filter and transferred to sample vials for subsequent UPLC–MS/MS analysis. Metabolite extraction, detection, and quantitative analysis were performed by MetWare Biotechnology Co., Ltd. (Wuhan, China; https://www.metware.cn/). A pooled quality-control (QC) sample was prepared by mixing aliquots of all sample extracts and was used to assess the repeatability of sample preparation and the stability of the LC–MS/MS analysis.

### 2.8. UPLC and Tandem Mass Spectrometry Conditions

Chromatographic separation was performed on an ultra-performance liquid chromatography system (ExionLC™ AD, SCIEX, Framingham, MA, USA) coupled with tandem mass spectrometry. An Agilent SB-C18 column (1.8 µm, 2.1 mm × 100 mm) was used. The mobile phases consisted of ultrapure water containing 0.1% (*v*/*v*) formic acid (A) and acetonitrile containing 0.1% (*v*/*v*) formic acid (B). The column temperature was set at 40 °C, the flow rate was 0.35 mL min^−1^, and the injection volume was 2 µL. Mass spectrometry was conducted using an electrospray ionization (ESI) source with a source temperature of 500 °C. The ion spray voltage was 5500 V in positive mode and −4500 V in negative mode. Gas settings were as follows: gas source I (GSI) 50 psi, gas source II (GSII) 60 psi, and curtain gas (CUR) 25 psi. Collision-induced dissociation was set to high, and multiple reaction monitoring (MRM) was used on a triple quadrupole (QQQ) mass analyzer (AB SCIEX QTRAP 4500, SCIEX, Framingham, MA, USA). Nitrogen was used as the collision gas with medium intensity. Declustering potential (DP) and collision energy (CE) were optimized for each MRM transition, and a set of specific MRM ion pairs was monitored according to metabolite elution during each analytical run.

### 2.9. Metabolite Identification and Quantification

Metabolites were identified based on the MetWare in-house database (MWDB; MetWare database) using MS/MS information [18]. During identification, isotopic signals, redundant signals corresponding to K^+^, Na^+^, and NH_4_^+^ adducts, and redundant fragment-ion signals derived from larger molecules were removed. Metabolite quantification was performed using the MRM mode on the triple quadrupole mass spectrometer, and mass spectrometric data were acquired for all samples. Peak areas were integrated for all detected metabolites, and peak integration was corrected across samples for the same metabolite. Metabolites were identified using processed peaks by searching a proprietary database from the Meteville laboratory and integrating public libraries, predicted libraries, and the MetDNA method [19]. Compounds were selected using an identification composite score < 0.5 and a coefficient of variation (CV) < 0.3 in quality control (QC) samples. Positive and negative ion modes were combined to retain compounds with the highest confidence and lowest CV values, thereby generating a comprehensive data file for all samples. Metabolites were characterized using both primary and secondary MS data. Relative levels of metabolites in each sample were determined based on chromatographic peak areas.

### 2.10. HPLC Quantification and Validation

An HPLC system (Hitachi L-2000, Tokyo, Japan) equipped with a pump (L-2130), an autosampler (L-2200), a column oven (L-2300), and a UV detector (L-2400, Hitachi, Tokyo, Japan) was used for analysis. Separation was performed on a GRACE Apollo C18 column (250 mm × 4.6 mm, 5 μm; Thermo Fisher Scientific, Waltham, MA, USA). The mobile phases consisted of methanol (A) and 0.5% (*v*/*v*) formic acid in water (B), with the following gradient program: 0 min, A/B = 20%/80% (*v*/*v*); 5 min, 35%/65%; 20 min, 50%/50%; 30 min, 70%/30%; 32 min, returned to 20%/80% and held until 35 min for column re-equilibration. The flow rate was set at 1.0 mL/min from 0–20 min, 0.8 mL/min from 20–30 min, and returned to 1.0 mL/min from 30–35 min. The detection wavelength was 325 nm, the column temperature was maintained at 30 °C, and the injection volume was 10 μL. External standard (purity ≥ 98%; Chengdu Zhibiao Chemical Co., Chengdu, China) solutions were prepared by dissolving each standard in methanol. For each standard, five concentration levels were used to construct calibration curves based on peak area versus concentration for quantification. Bamboo leaf extracts were filtered through a 0.22 μm membrane prior to injection. Target compounds were identified by matching retention times with the corresponding standards and quantified using the external standard method.

### 2.11. Data Processing and Statistical Analysis

Data were organized in Excel 2024 and uploaded to the MetWare Cloud platform (https://cloud.metware.cn/#/user/login, accessed on 10 June 2025) for principal component analysis (PCA), hierarchical cluster analysis (HCA), and orthogonal partial least squares-discriminant analysis (OPLS-DA). Differentially accumulated metabolites among the three altitudes were defined using a variable importance in projection (VIP) ≥1 and a fold change (FC) ≥2 or ≤0.5. Functional annotation and classification of differentially accumulated metabolites were performed using the Kyoto Encyclopedia of Genes and Genomes (KEGG) database. Statistical significance was assessed by one-way analysis of variance (ANOVA), followed by Tukey’s test at *p* < 0.05. Unless otherwise stated, the data for total flavonoid content, total phenolic content, DPPH radical-scavenging activity, ABTS radical-scavenging activity, reducing power, and HPLC quantification are presented as mean ± standard deviation (SD) based on three independent biological replicates (*n* = 3). Statistical significance among the three altitude groups was evaluated by one-way analysis of variance (ANOVA) followed by Tukey’s multiple comparison test. Differences were considered significant at *p* < 0.05, and bars labeled with different letters in the figures indicate statistically significant differences among groups. Pearson correlation analysis was used to assess the relationships between differential flavonoids and antioxidant indices, and correlations with *p* < 0.05 were considered statistically significant unless otherwise stated.

## 3. Results

### 3.1. Polyphenol Contents, Soluble Sugar Content and In Vitro Antioxidant Capacity of Chimonobambusa utilis Leaf Extract Across Altitudes

**Figure 1:**
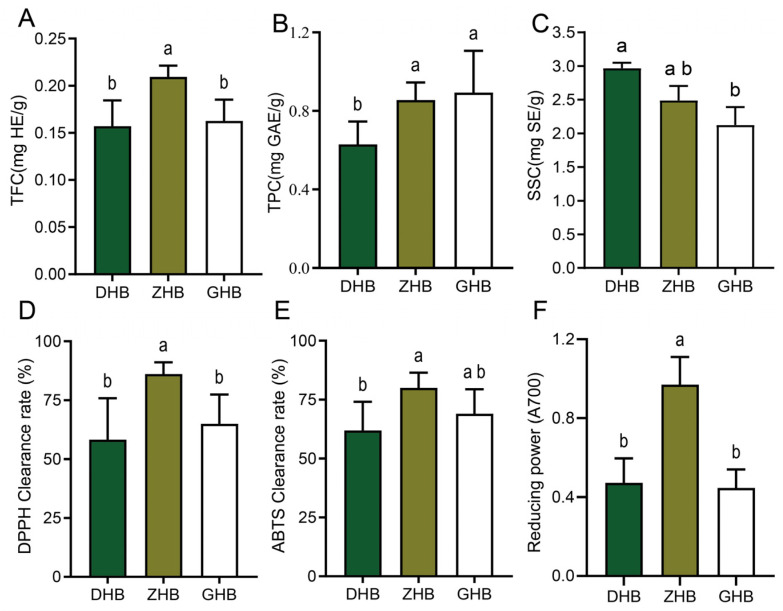
Total flavonoid content, total phenolic content, soluble sugar content and antioxidant activities of *Chimonobambusa utilis* leaves across three elevation groups (DHB, ZHB and GHB). (**A**) Total flavonoid content (TFC, mg HE/g). (**B**) Total phenolic content (TPC, mg GAE/g). (**C**) Soluble sugar content (SSC, mg SE/g DW). (**D**) DPPH radical scavenging activity. (**E**) ABTS radical scavenging activity. (**F**) Reducing power expressed as absorbance at 700 nm (A700). Data are presented as mean ± SD (*n* = 3). Different letters above bars indicate statistically significant differences among groups as determined by one-way ANOVA followed by Tukey’s multiple comparison test (*p* < 0.05). HE, hyperoside equivalents; GAE, gallic acid equivalents; SE, sucrose equivalents.

Across the three altitude groups, total flavonoid content (TFC, mg HE/g) was highest in the mid-altitude group (ZHB, 0.210), significantly exceeding that in the low-altitude group (DHB, 0.157) and the high-altitude group (GHB, 0.163); no significant difference was detected between DHB and GHB (Figure 1A). Total phenolic content (TPC, mg GAE/g) was higher in ZHB (0.856) and GHB (0.893) than in DHB (0.630), with no significant difference between ZHB and GHB (Figure 1B). In contrast, soluble sugar content (SSC, mg SE/g DW) was highest in DHB (2.998), followed by ZHB (2.488), and lowest in GHB (2.125). SSC in DHB was significantly higher than that in GHB, whereas no significant difference was detected between DHB and ZHB (Figure 1C).

Regarding in vitro antioxidant activity, ZHB exhibited stronger overall redox capacity (Figure 1D–F). Specifically, DPPH radical-scavenging activity reached 86.1% in ZHB, which was significantly higher than in DHB (58.3%) and GHB (65.0%) (Figure 1D). ABTS radical-scavenging activity in ZHB reached 79.9%, significantly higher than in DHB (62.0%), whereas GHB showed an intermediate level (69.0%) (Figure 1E). Reducing power (A700) was also highest in ZHB (0.970), significantly exceeding that in DHB (0.488) and GHB (0.445) (Figure 1F). Overall, all three antioxidant indices peaked in ZHB, consistent with the higher TFC at this altitude, whereas SSC showed an opposite trend and was highest in DHB.

### 3.2. Quality Control of Widely Targeted Metabolomics Data for Ch. utilis Leaves

**Figure 2:**
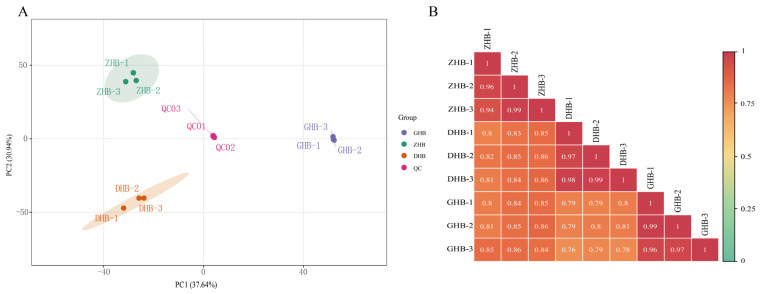
Principal component analysis (PCA) and sample-to-sample correlation of metabolite profiles from *Chimonobambusa utilis* leaves collected at three elevations (DHB, ZHB and GHB). (**A**) PCA score plot of DHB, ZHB, GHB and pooled quality-control (QC) samples; each point represents one replicate, and shaded ellipses indicate the 95% confidence region for each group. (**B**) Pearson correlation heatmap based on metabolite peak areas; colors and numbers indicate Pearson’s correlation coefficients (*r*). All pairwise correlations shown in (**B**) were statistically significant (*p* < 0.01).

Widely targeted metabolomics based on LC–MS/MS was employed to profile metabolites in *Ch. utilis* leaves collected from three altitude groups (DHB, ZHB, and GHB), and data quality was evaluated in terms of method repeatability and instrument stability prior to downstream analyses. The total ion chromatograms (TICs) of the QC samples showed overall consistent peak shapes and intensities across injections, indicating good reproducibility of sample preparation and detection. Principal component analysis (PCA) showed that PC1 and PC2 explained 37.64% and 30.94% of the variance, respectively, with a cumulative contribution of 68.58% (Figure 2A). In widely targeted metabolomics datasets, where thousands of metabolites contribute to sample variation, the first two principal components do not necessarily capture all of the total variance. Nevertheless, the clear separation among the three altitude groups, the tight clustering of biological replicates within each group, and the close aggregation of QC samples indicate that the PCA still effectively reflects the major variation pattern and supports the reliability of the metabolomic dataset. Pearson correlation analysis further showed high within-group correlations (*r* ≥ 0.94) and relatively high between-group correlations (*r* ≥ 0.76) (Figure 2B), confirming reliable sample grouping.

### 3.3. Metabolite Profile of Ch. utilis Leaves Across Altitudes

**Figure 3:**
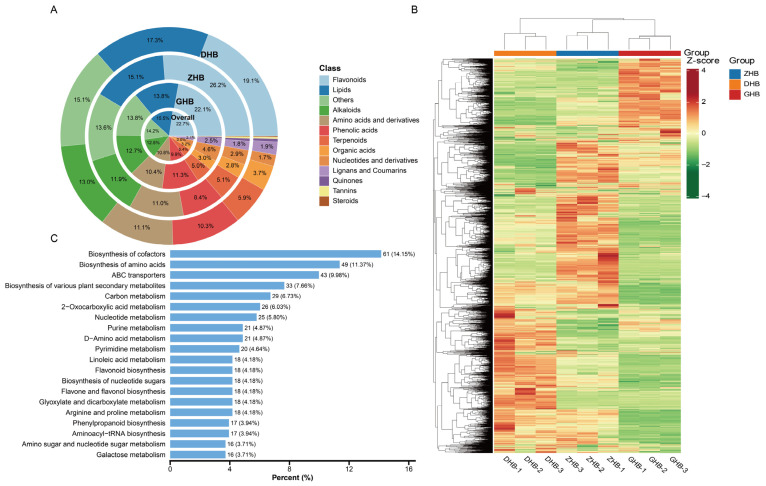
Overview of the metabolome, chemical class composition and KEGG functional annotation of *Chimonobambusa utilis* leaves across the three elevation groups. (**A**) Chemical class composition shown as a concentric doughnut chart; from inner to outer rings: overall, GHB, ZHB and DHB, with numbers indicating the percentage contribution of each class. (**B**) Heatmap with hierarchical clustering of metabolite profiles across samples; values are Z-score normalized for each metabolite across samples. (**C**) KEGG pathway classification of annotated metabolites (Top 20); the x-axis indicates the percentage of metabolites mapped to each pathway, and bar-end labels show metabolite counts with the corresponding proportions.

A total of 3113 metabolites were detected in *Ch. utilis* leaves and assigned to 13 major compound classes. Overall, flavonoids accounted for the largest proportion of relative abundance (22.7%), followed by lipids (15.5%), alkaloids (12.5%), amino acids and derivatives (10.8%), and phenolic acids (9.9%) (Figure 3A). Flavonoids were the dominant class in all three altitude groups, with the highest proportion in ZHB (26.2%), exceeding that in DHB (19.1%) and GHB (22.1%); in contrast, phenolic acids showed the highest proportion in GHB (11.3%).

The hierarchical clustering heatmap (Figure 3B), constructed using Z-score–normalized abundances of all 3113 metabolites, showed tight clustering of biological replicates within each altitude group and clear separation among groups. Combined with class (trend) analysis, metabolites were grouped into three classes according to their standardized accumulation patterns across DHB, ZHB, and GHB (Figure S1). Class 1 contained 1228 metabolites with relatively higher levels in ZHB, class 2 contained 763 metabolites with relatively higher levels in GHB, and class 3 contained 1122 metabolites with relatively higher levels in DHB. Within class 1, flavonoids were the most represented compound class (295 metabolites, 24.02%), and their summed relative abundance accounted for 34.34% of the total relative abundance within class 1, higher than that in class 2 (145 metabolites, 19.00%; 20.40%) and class 3 (164 metabolites, 14.62%; 21.18%). Collectively, these results indicate that flavonoids are dominant in both number and overall abundance within class 1, which shows relatively higher levels in ZHB.

KEGG annotation showed that 431 of the 3113 metabolites were assigned to 107 KEGG pathways. As shown in (Figure 3C), pathways directly related to flavonoid and polyphenol metabolism mainly included Biosynthesis of various plant secondary metabolites (33 metabolites, 7.66%), Flavonoid biosynthesis (18 metabolites, 4.18%), Flavone and flavonol biosynthesis (18 metabolites, 4.18%), and Phenylpropanoid biosynthesis (17 metabolites, 3.94%). In total, these four pathways involved 86 metabolites, accounting for 19.96% of the KEGG-annotated metabolites, indicating that flavonoid- and polyphenol-related biosynthetic processes represent a substantial part of the overall metabolic network.

### 3.4. Differential Metabolite Analysis of Ch. utilis Leaves Across Altitudes

**Figure 4:**
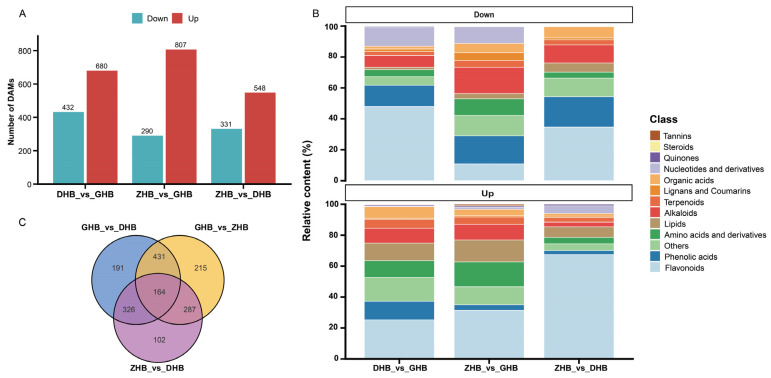
Statistics, class composition and overlap of differentially accumulated metabolites (DAMs) in *Chimonobambusa utilis* leaves along the elevational gradient. (**A**) Numbers of up and down accumulated DAMs in the pairwise comparisons (DHB_vs_GHB, ZHB_vs_GHB and ZHB_vs_DHB); Up and Down indicate higher and lower abundance in the first group of each comparison, respectively. DAMs were defined using fold change (FC ≥ 2 or FC ≤ 0.5) and variable importance in projection (VIP ≥ 1), with VIP values derived from the OPLS-DA model. (**B**) Chemical class composition of DAMs based on their summed relative contents (%, calculated from metabolite peak areas), shown separately for up- and down-accumulated metabolites. (**C**) Venn diagram showing shared and unique DAMs among the three pairwise comparisons.

Pairwise comparisons among the three elevation groups identified a total of 1716 differentially accumulated metabolites (non-redundant DAMs across all pairwise comparisons), including 423 flavonoid DAMs. “Up” and “Down” denote DAMs that were higher/lower in the former group than in the latter: DHB_vs_GHB yielded 1112 DAMs (Up 680, Down 432), ZHB_vs_GHB yielded 1097 DAMs (Up 807, Down 290), and ZHB_vs_DHB yielded 879 DAMs (Up 548, Down 331) (Figure 4A).

As shown in (Figure 4B) and (Table S1), the class-wise composition of up- and downregulated DAMs was summarized based on relative content (%). In the upregulated sets, flavonoids were the major class and their relative-content share was highest in ZHB_vs_DHB (67.46%; 222 flavonoids). Flavonoids also remained the leading class in the upregulated sets of ZHB_vs_GHB and DHB_vs_GHB (31.37%, 168 flavonoids; and 25.23%, 116 flavonoids, respectively). In contrast, the class composition of downregulated DAMs differed more markedly among comparisons. Flavonoids still dominated the downregulated set in DHB_vs_GHB (47.96%, 151 flavonoids), whereas in ZHB_vs_GHB, phenolic acids and alkaloids accounted for larger shares of the relative content (18.15% and 16.99%, respectively). Overall, comparisons involving ZHB showed a more pronounced flavonoid enrichment within the upregulated DAMs.

Venn analysis revealed 164 shared DAMs across the three pairwise comparisons (Figure 4C), with flavonoids (58) being the most abundant class, followed by phenolic acids (29), indicating that the shared DAMs were mainly composed of polyphenols. Among the 58 shared differential flavonoids, 33 reached their highest mean relative abundances in ZHB, showing 2.01–210.02 fold higher mean relative abundances than DHB and 2.33–61.54 fold higher than GHB. Glycosylation-type profiling indicated that these ZHB-peaking flavonoids were mainly O-glycosides (19), followed by C-glycosides (9) (Table S2).

### 3.5. KEGG Enrichment Analysis of Differential Metabolites Among Altitude Groups

**Figure 5:**
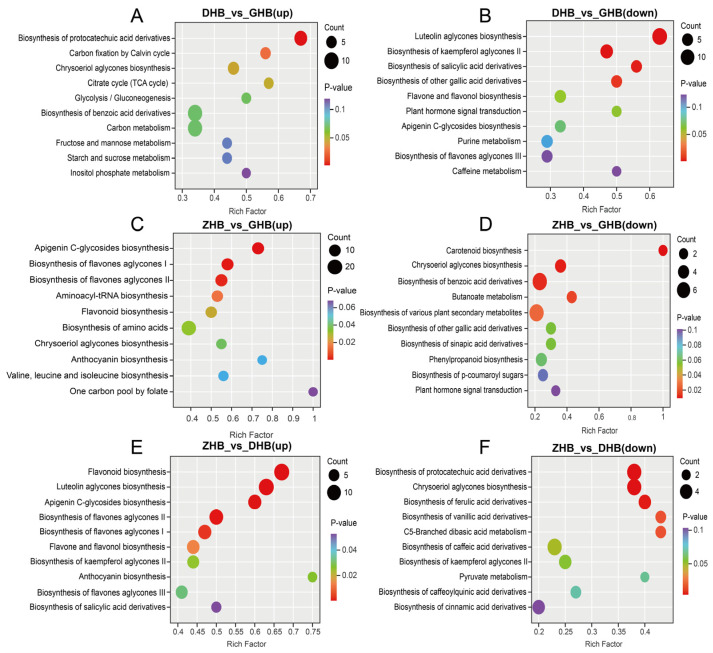
Top 10 enriched KEGG pathways of upregulated and downregulated differential metabolites in pairwise comparisons among DHB, ZHB and GHB. (**A**) DHB vs. GHB (up); (**B**) DHB vs. GHB (down); (**C**) ZHB vs. GHB (up); (**D**) ZHB vs. GHB (down); (**E**) ZHB vs. DHB (up); (**F**) ZHB vs. DHB (down). Bubble size indicates the number of enriched differential metabolites, and bubble color indicates the *p*-value.

KEGG enrichment analysis of pairwise comparisons among the three altitude groups (Figure 5) showed that, in the DHB vs. GHB comparison, the top enriched pathways included Carbon fixation by Calvin cycle, Carbon metabolism, Glycolysis/Gluconeogenesis, Citrate cycle (TCA cycle), Biosynthesis of protocatechuic acid derivatives, and Biosynthesis of benzoic acid derivatives (Figure 5A,B). In the ZHB vs. GHB comparison, the top enriched pathways included Apigenin C-glycosides biosynthesis, Biosynthesis of flavones aglycones I and II, Flavonoid biosynthesis, Anthocyanin biosynthesis, Biosynthesis of amino acids, Aminoacyl-tRNA biosynthesis, and One carbon pool by folate (Figure 5C,D). In the ZHB vs. DHB comparison, the top enriched pathways included Flavonoid biosynthesis, Flavone and flavonol biosynthesis, Luteolin aglycones biosynthesis, Apigenin C-glycosides biosynthesis, Anthocyanin biosynthesis, and several phenolic acid-related pathways (Figure 5E,F).

### 3.6. Flavonoid Variation in Ch. utilis Leaves Across Elevations

**Figure 6:**
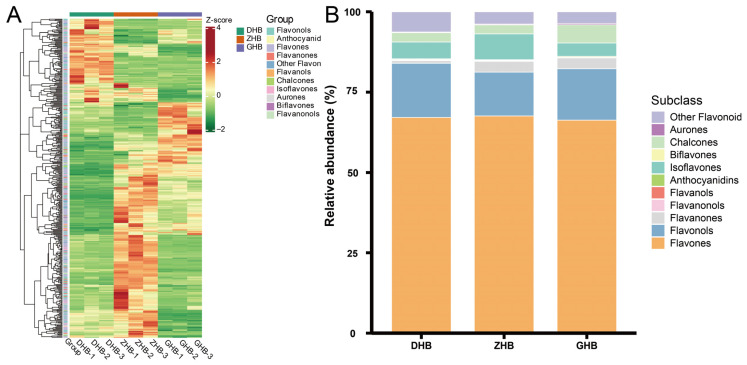
Accumulation patterns and subclass composition of differentially accumulated flavonoids (DAFs) in *Chimonobambusa utilis* leaves across the three elevation groups. (**A**) Hierarchical clustering heatmap of 423 DAFs; values are Z-score normalized for each flavonoid across samples. (**B**) Relative contribution (%) of flavonoid subclasses in DHB, ZHB and GHB, calculated from the summed relative contents (peak areas) of DAFs.

The hierarchical clustering heatmap based on the 423 differential flavonoids showed clear separation in flavonoid accumulation patterns among the three elevation groups (Figure 6A). Further trend-based classification grouped these 423 differential flavonoids into eight accumulation patterns (Sub class 1–8) (Figure S2; Table S3). Sub class 2, 3, 7, and 8 displayed a typical ZHB-peaking pattern (increasing from DHB to ZHB, reaching the maximum at ZHB, and then declining toward GHB), comprising 258 flavonoids in total (36, 66, 52, and 104, respectively). In contrast, Sub class 1 and Sub class 6 generally increased with elevation (higher in GHB), Sub class 4 decreased with elevation (higher in DHB), and Sub class 5 showed a trough at ZHB. In addition, within ZHB, the cumulative relative content of these 258 differential flavonoids accounted for 83.01% of the cumulative relative content of all 423 differential flavonoids. Collectively, these results indicate that ZHB shows both higher flavonoid richness and higher relative content, representing the key elevation interval driving flavonoid accumulation differences in *Ch. utilis* leaves.

Further subclass profiling showed that flavones dominated across all three groups (Figure 6B; Table S3), accounting for 66.29–67.60% of the relative content, with the highest proportion in ZHB (67.60%). Meanwhile, isoflavones accounted for 8.02% in ZHB, higher than in DHB (5.20%) and GHB (4.11%), whereas flavonols represented a lower proportion in ZHB (13.61%) than in DHB (16.84%) and GHB (15.98%).

### 3.7. Correlation Network Reveals Key Flavonoids Associated with Antioxidant Capacity

**Figure 7:**
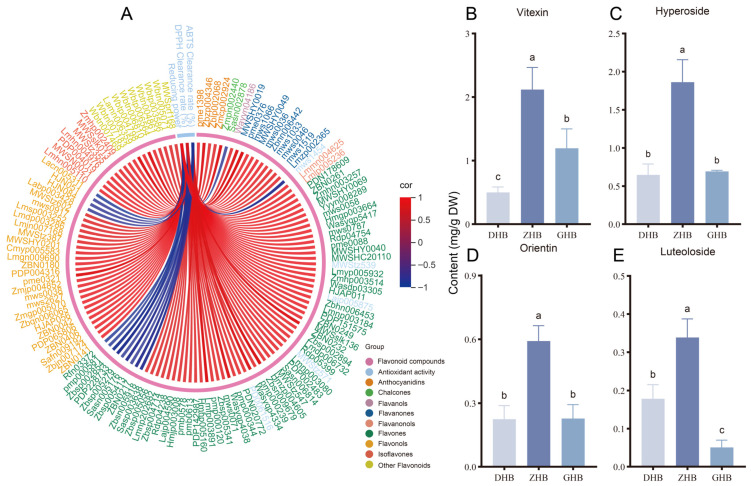
(**A**) Pearson correlation chord diagram linking 423 differential flavonoids with antioxidant indices (DPPH radical scavenging activity, ABTS radical scavenging activity and reducing power at 700 nm, A700). Only significant correlations are shown (|r| > 0.8, *p* < 0.05). Red and blue links indicate positive and negative correlations, respectively. HPLC quantification of (**B**) vitexin, (**C**) hyperoside, (**D**) orientin, and (**E**) luteoloside in leaves collected from three elevation groups (DHB, ZHB, and GHB). Data are presented as mean ± SD (*n* = 3). Different letters above bars indicate statistically significant differences among elevation groups as determined by one-way ANOVA followed by Tukey’s multiple comparison test (*p* < 0.05).

Pearson correlation analysis was performed between the 423 differential flavonoids and three antioxidant indices (DPPH and ABTS scavenging rates, and total reducing power), and an association network was constructed. Under the screening threshold (*r* > 0.8, *p* < 0.05), a total of 115 flavonoids were identified that were significantly and positively correlated with at least one antioxidant index (Figure 7A). The relative-content proportions reported below were calculated within the significantly correlated flavonoid set for each index (i.e., using the cumulative relative content of that set as the denominator). Structural feature statistics further showed that 113 flavonoids significantly promoted total reducing power, including 99 flavonoids with a conjugated scaffold containing a C2 = C3 double bond (relative-content proportion, 98.42%), 41 flavonoids with ≥2 hydroxyl groups on the B-ring (mainly dihydroxy; relative-content proportion, 39.06%), and 77 glycosylated flavonoids with a relatively high relative-content proportion (75.25%) (Table S4). A total of 10 flavonoids were significantly and positively correlated with the DPPH scavenging rate, with major contributions from flavonoids with a conjugated C2 = C3 scaffold (7; relative-content proportion, 55.27%) and O-glycosylated flavonoids (5; relative-content proportion, 75.45%). A total of 8 flavonoids were significantly and positively correlated with the ABTS scavenging rate, in which B-ring dihydroxy flavonoids (3; relative-content proportion, 61.10%) and flavonoids with a conjugated C2 = C3 scaffold (5; 61.34%) accounted for high relative-content proportions, suggesting that ABTS scavenging may be dominated by a small number of high-abundance flavonoids (Table S4).

### 3.8. Quantitative Validation of Representative Flavonoids Using HPLC

To provide biochemical quantitative validation for the metabolomics-derived flavonoid accumulation pattern, four representative flavonoids, vitexin, hyperoside, orientin, and luteoloside, were selected for HPLC quantification from the 33 flavonoids enriched in the ZHB group. These compounds were chosen not solely based on VIP values, but also based on their clear mid-altitude enrichment pattern, structural representativeness among the major ZHB-enriched flavonoid subclasses, and the availability of authentic commercial standards. Specifically, they represented apigenin-type C-glycosides, luteolin-type C/O-glycosides, and quercetin-type flavonol glycosides, as reflected by the high fold changes in Apigenin-6-C-arabinoside-4′-O-xyloside (ZHB/DHB = 6.67, ZHB/GHB = 61.54), Orientin 2″-O-β-L-arabinofuranoside (6.40, 58.88), Isovitexin-8-O-xyloside (6.23, 44.57), Luteolin-7-O-(6″-sinapoyl)glucoside (4.72, 21.08), and Quercetin-3-O-alloside/Isohyperoside (6.45, 2.98). The calibration curves showed good linearity (R^2^ = 0.999), and the precision, repeatability, and stability tests all showed RSD values below 5%.

HPLC results indicated that all four flavonoid monomers differed significantly among the three elevations and reached their highest contents in ZHB (Figure 7B–E). Specifically, vitexin reached 2.12 mg g^−1^ DW in ZHB, which was 4.24-fold and 1.78-fold higher than in DHB and GHB, respectively. Hyperoside reached 1.86 mg g^−1^ DW in ZHB, corresponding to 2.86-fold and 2.70-fold increases relative to DHB and GHB, respectively. Orientin reached 0.59 mg g^−1^ DW in ZHB, representing 2.68-fold and 2.57-fold increases relative to DHB and GHB, respectively. Luteoloside reached 0.34 mg g^−1^ DW in ZHB, which was 1.89-fold higher than in DHB and 6.80-fold higher than in GHB. Overall, the HPLC quantification results were consistent with the metabolomics-based inference of higher flavonoid levels in ZHB.

## 4. Discussion

### 4.1. Metabolic Pathway Remodeling and the Growth-Defense Trade-Off in Chimonobambusa utilis Leaves Along an Altitudinal Gradient

Altitudinal gradients represent a complex composite of environmental stressors, including temperature fluctuations and varying UV-B irradiance [20]. For *Ch. utilis*, adapting to these gradients requires a constant reallocation of metabolic resources, perfectly illustrating the “growth-defense trade-off” theory [21]. This theory posits that plants must dynamically balance limited energy and carbon resources between primary metabolism (growth) and secondary metabolism (defense and stress acclimation). Our metabolomic profiling reveals that altitude reshapes the leaf metabolome not merely by inducing single pathways, but by fundamentally shifting this resource allocation.

Low Elevation (1150 m, DHB)—Prioritizing Primary Growth: At lower altitudes, milder environmental conditions allow *Ch. utilis* to prioritize growth. KEGG enrichment indicates that the DHB group is predominantly associated with primary carbon assimilation, including the Calvin cycle, glycolysis, and the TCA cycle (Figure 5A). While these primary pathways actively generate abundant ATP and essential carbon skeletons (such as the aromatic amino acid precursors required for plant growth) [22], the relatively low environmental stress is insufficient to channel significant metabolic flux downstream into the defense-oriented phenylpropanoid–flavonoid network. For instance, under non-stressful conditions, plants preferentially commit carbon resources to biomass accumulation and continuous protein synthesis. Without strong environmental elicitors—such as severe temperature drops or intense UV radiation—to trigger stress-signaling cascades, crucial enzymatic bottlenecks (e.g., phenylalanine ammonia-lyase) remain largely unactivated, thereby restricting the carbon flux from entering the secondary defense metabolome [23,24]. Consequently, a large proportion of metabolic flux remains in primary and upstream phenolic pathways, resulting in a lower relative proportion of complex flavonoids. In addition, the low-altitude group showed the highest soluble sugar content (Figure 1C), and several representative sugar-related metabolites, including D-glucose, D-fructose, D-mannose, D-galactose, and raffinose, also exhibited relatively higher abundance in DHB (Table S5). These results provide complementary support for relatively stronger primary carbon metabolism-related characteristics at low altitude.

Mid Elevation (1600 m, ZHB)—The Optimal Trade-off (The “Sweet Spot”): The highest total flavonoid content and the most robust antioxidant capacity are strictly localized to the mid-elevation. This occurs because 1600 m represents an optimal metabolic “sweet spot.” Here, the moderate environmental stress serves as a potent elicitor, fully upregulating the phenylpropanoid and flavonoid biosynthetic pathways, which are critical for stress tolerance and redox regulation (Figure 5C,E). For instance, parallel altitudinal studies on *Coffea arabica* and *Zanthoxylum planispinum* have demonstrated that mid-altitude environments provide a similar optimal balance: the moderate temperature and light cues are sufficient to trigger stress-signaling and upregulate defense-related polyphenols, yet they do not cause the severe photosynthetic and carbon-assimilation inhibition typically observed at extreme alpine elevations [22,25,26]. Crucially, unlike the extreme GHB environment, the moderate ZHB environment does not severely suppress photosynthesis; it allows the plant to maintain a steady upstream supply of amino acids and carbon precursors. This successful coupling—where sustained primary metabolic supply directly feeds highly induced secondary metabolic flux—enables the maximum accumulation of flavonoids. This coordinated carbon allocation under moderate stress is widely recognized as a key driver of chemical diversity and physiological acclimation in plants.

Similar non-linear, mid-elevation advantages for secondary metabolite accumulation have been widely reported in other species. For instance, recent studies on *Zanthoxylum planispinum*, Turkish hazelnut cultivars, and *Coffea arabica* consistently demonstrate that flavonoid levels and associated antioxidant activities peak at mid-altitudes [8,25,26,27]. In bamboos specifically, altitudinal variation has been proven to significantly alter leaf chemical composition and reshape the distribution patterns of key bioactive flavonoids, such as vitexin and isovitexin, concurrently driving functional divergence in leaf redox capacity [9]. Ultimately, the mid-elevation environment provides the perfect balance of induction intensity and metabolic capacity, driving the superior flavonoid diversity and antioxidant phenotype observed in *Ch. utilis* leaves.

### 4.2. Structural-Activity Basis of Flavonoid Antioxidative Plasticity in Ch. utilis Leaves

Altitudinal variation can alter the accumulation patterns of secondary metabolites in plant tissues, thereby influencing plant adaptation to environmental change [28]. With increasing altitude, elevated levels of reactive oxygen species (ROS) in plants may damage cellular membrane systems, leading to metabolic disorders and even cell death [29]. Antioxidant activity in plant extracts is commonly evaluated by DPPH radical-scavenging activity, ABTS radical-scavenging activity, and total reducing power [30]. In the present study, leaf extracts of *Ch. utilis* from the ZHB group exhibited relatively high DPPH and ABTS radical-scavenging activities as well as strong total reducing power. Furthermore, correlation analysis and network construction based on 423 differential flavonoids identified by widely targeted metabolomics and three redox-related indices revealed 115 flavonoids positively associated with enhanced redox capacity, all of which were enriched in ZHB. These results suggest that flavonoids enriched at mid-altitudes may possess structural features more favorable for redox reactions, thereby making an important contribution to the stronger redox capacity observed in ZHB. This finding further indicates that differences in the redox phenotype of *Ch. utilis* leaves are associated not only with the level of flavonoid accumulation, but also potentially with the specific structural characteristics of flavonoid molecules.

Flavonoids are considered one of the most important natural antioxidants in plants [31], which is consistent with our observation that ZHB showed higher flavonoid accumulation and stronger antioxidant capacity in bamboo leaf extracts. In vitro antioxidant activity of flavonoids is often governed by key structural elements. Increased B-ring hydroxylation generally enhances radical-scavenging capacity [32]. In particular, the catechol motif can stabilize the phenoxyl radical via electron delocalization, thereby strengthening antioxidant activity [33]. Meanwhile, the conjugated system formed by the C2 = C3 double bond and the 4-oxo group in the C-ring can markedly enhance electron delocalization and stabilize radical intermediates, leading to improved in vitro scavenging capacity; by contrast, flavanones lacking this conjugation often show weaker activity [34].

The Pearson correlation network further indicated clear structure-related preferences in the contributions of differential flavonoids to antioxidant indices. First, total reducing power is a typical single-electron transfer (SET/ET) assay, mainly reflecting the ability of flavonoids to transfer electrons to metal-oxidant systems and complete reduction, and therefore better represents the overall capacity of flavonoids as electron donors [35]. Compared with the DPPH and ABTS systems, total reducing power more readily amplifies structural combinations characterized by a conjugated scaffold plus multiple hydroxyl-based electron-donating sites. In this study, flavonoids significantly associated with total reducing power were dominated by flavone- and flavonol-type backbones retaining the C2 = C3–4-oxo conjugation. This conjugated system enhances π-electron delocalization and stabilizes oxidized radical intermediates, thereby improving sustained electron-donating efficiency and reducing performance [36]. In addition, modifications such as glycosylation and acylation may modulate ET performance by altering polarity, reaction accessibility, and the extent of delocalization. For example, juglanin (kaempferol-3-O-arabinoside) has been suggested to retain the conjugated kaempferol scaffold while increasing aqueous-phase accessibility, thereby enhancing Fe^3+^-reducing capacity [37].

ABTS scavenging is more closely related to electron donation from flavonoids to ABTS•^+^. After generation, ABTS•^+^ can react with antioxidants in both aqueous and organic phases, making it more suitable for reflecting overall antioxidant capacity [38]. Mechanistically, ABTS is often regarded as a SET-dominated system; under aqueous conditions, it may be reduced via pathways such as SPLET (sequential proton loss electron transfer), and is therefore more sensitive to phenolic hydroxyl structures that can stabilize radical intermediates and show stronger electron delocalization [39]. In this study, flavonoids significantly and positively correlated with ABTS scavenging more often featured both B-ring catechol-type dihydroxylation and a conjugated scaffold, indicating that the ABTS assay can capture contributions from compounds across a wide polarity range. Structure–activity evidence suggests that ABTS•^+^ is more sensitive to electron delocalization capacity: catechol-type dihydroxylation promotes resonance stabilization of phenoxyl radicals, while C-ring conjugation can further extend delocalization, and the two often form a core structural combination for efficient scavenging [40].

DPPH scavenging showed pronounced kinetic and accessibility filtering. As a relatively hydrophobic and stable radical, DPPH has limited effective contact with hydrophilic flavonoids [41]. Its readout depends not only on thermodynamic feasibility but is more readily amplified by early reaction rates and HAT kinetics [42], and steric effects that alter access to reactive sites can further influence scavenging capacity [43]. Accordingly, DPPH is highly sensitive to initial reaction rates and steric accessibility, often highlighting structural groups that react efficiently under assay conditions and rapidly undergo hydrogen or electron transfer. Consistent with this, flavonoids associated with DPPH scavenging in this study tended to show lower B-ring hydroxylation, with prominent contributions from O-glycosides (e.g., chrysoeriol-7-*O*-rutinoside and kaempferol O-glycosides). The set also included compounds with relatively fast H-donor features, such as neohesperidin and eriodictyol chalcone. Previous studies have likewise reported that some O-glycosylated flavonoids (e.g., rutin and chrysoeriol 7-*O*-rutinoside) exhibit strong scavenging activity in the DPPH system [44].

Finally, absolute quantification of four representative flavonoids was performed by HPLC. The results showed that vitexin, hyperoside, orientin, and luteoloside all reached their highest absolute contents in ZHB, exhibiting a highly consistent ZHB-peaking accumulation pattern. This trend closely matched the overall superior redox-related functional performance of ZHB extracts, providing robust quantitative biochemical evidence for the specific enrichment of the mid-elevation flavonoid chemotype. From a structural and biochemical perspective, these four flavonoids collectively cover the key structural elements discussed above. Orientin and luteoloside share a luteolin aglycone, retain the catechol motif on the B-ring, and are coupled with the C-ring conjugated system. This combination is thought to enhance electron delocalization and stabilize radical intermediates, thereby favoring activity in predominantly single-electron transfer (SET) systems such as ABTS and total reducing power [45,46]. Hyperoside, a typical flavonol glycoside, features a higher degree of hydroxylation and stronger resonance-stabilization potential, providing a structural basis for sustained electron donation and reducing capacity [47]. Vitexin, as a C-glycosyl flavone, is structurally more stable and less prone to degradation, and thus may serve as a baseline redox-buffering component that can be maintained in leaves [48]. Overall, HPLC-based quantification not only strengthens the mid-elevation enrichment pattern revealed by metabolomics, but also provides direct chemical evidence supporting the structure-selective preferences observed across different antioxidant assays.

### 4.3. Study Limitations and Future Perspectives

Despite the clear altitude-associated differences observed in this study, several limitations should be acknowledged. The samples were collected from a single mountain range during a single season, and the current conclusions therefore require further validation across broader spatial and temporal scales. In addition, altitude was treated in this study as a composite ecological gradient rather than a single mechanistic factor. Other environmental parameters, such as light intensity and soil properties, were not directly measured. Therefore, the proposed explanation for enhanced flavonoid accumulation and antioxidant capacity at mid elevation should be regarded as an inference based on metabolomic patterns, antioxidant phenotypes, and published literature, rather than direct evidence of the effects of individual environmental factors. In addition, the antioxidant evidence was mainly derived from extract-based in vitro assays and metabolite–trait correlations, rather than from direct measurements of oxidative stress status in leaf tissues. For example, ROS accumulation and related antioxidant defense indicators were not assessed in the present study, and in vivo antioxidant effects were also not examined.

Future studies should validate the present findings using *Ch. utilis* samples collected from other mountain ranges and across different seasons, and could also test whether similar flavonoid remodeling patterns occur in other bamboo species along altitudinal gradients.

## 5. Conclusions

In conclusion, altitudinal variation markedly reshaped flavonoid accumulation in *Chimonobambusa utilis* leaves and was closely associated with variation in the antioxidant capacity of leaf extracts. Flavonoids were the dominant metabolites across all elevation groups, and the ZHB group exhibited a higher relative flavonoid level together with stronger DPPH and ABTS radical-scavenging activities and total reducing power in leaf extracts. Comparative pathway analysis further suggested that leaves from different elevations adopted distinct metabolic strategies, with ZHB showing a metabolic configuration more favorable for flavonoid biosynthesis and accumulation. Correlation analysis indicated that representative flavonoids enriched at mid elevation may constitute an important chemical basis for the enhanced extract-based antioxidant capacity. Overall, these findings link altitude-driven metabolic reprogramming with specific flavonoid variation and extract antioxidant capacity, providing new metabolomic evidence for understanding the physiological and ecological acclimation of this species along altitudinal gradients.

## Data Availability

The data presented in this study are available on request from the corresponding author.

## Supplementary Materials

The following supporting information can be downloaded at: https://www.mdpi.com/article/10.3390/plants15091290/s1, Figure S1: Calinski–Harabasz index-guided clustering grouped 3113 metabolites into three altitude-enriched clusters across DHB, ZHB and GHB; Figure S2: Trend-based clustering grouped 423 differentially accumulated flavonoids into eight accumulation subclasses across DHB, ZHB and GHB; Figure S3: Representative morphology of sampled *Chimonobambusa utilis* plants and leaves; Table S1: DAMs from pairwise comparisons among the three elevation groups; Table S2: Shared ZHB-enriched differential flavonoids (33); Table S3: ZHB-enriched differential flavonoids (423); Table S4: Flavonoids associated with three antioxidant indices; Table S5: Representative sugar-related metabolites enriched in DHB compared with ZHB and GHB.
