# Supplementary material for: Elevational Metabolic Reprogramming Optimizes Flavonoid Accumulation and Antioxidant Capacity in Chimonobambusa utilis Leaves
Source: Plants (Basel). 2026 Apr 22;15(9):1290. doi: 10.3390/plants15091290 (PMC13165436; doi:10.3390/plants15091290)
Supplement: Supplementary file 1 [file plants-15-01290-s001.zip › Supplementary Figure.pdf]

Elevational metabolic reprogramming optimizes flavonoid accumulation and antioxidant capacity in *Chimonobambusa utilis* leaves

Figure S1: Calinski–Harabasz index-guided clustering grouped 3,113 metabolites into three altitude-enriched clusters across DHB, ZHB and GHB

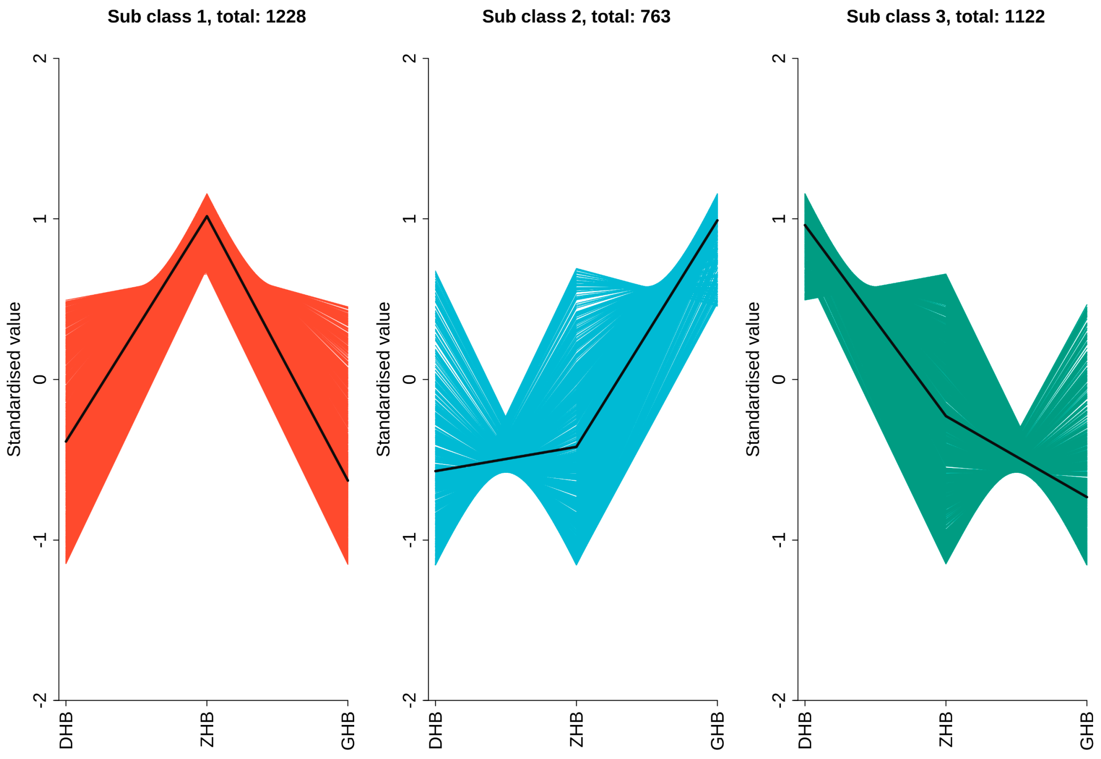

Figure. S1. Calinski–Harabasz index-guided clustering of altitude-enriched metabolites.Z-score standardised profiles of 3,113 metabolites across DHB, ZHB and GHB were grouped into three clusters (k = 3). Coloured lines represent individual metabolites and the black line indicates the mean trend. Cluster 1 (n = 1,228), Cluster 2 (n = 763) and Cluster 3 (n = 1,122) show enrichment in ZHB, GHB and DHB, respectively, consistent with the hierarchical clustering in Fig. 3B.

Figure S2: Trend-based clustering grouped 423 differentially accumulated flavonoids into eight accumulation subclasses across DHB, ZHB and GHB

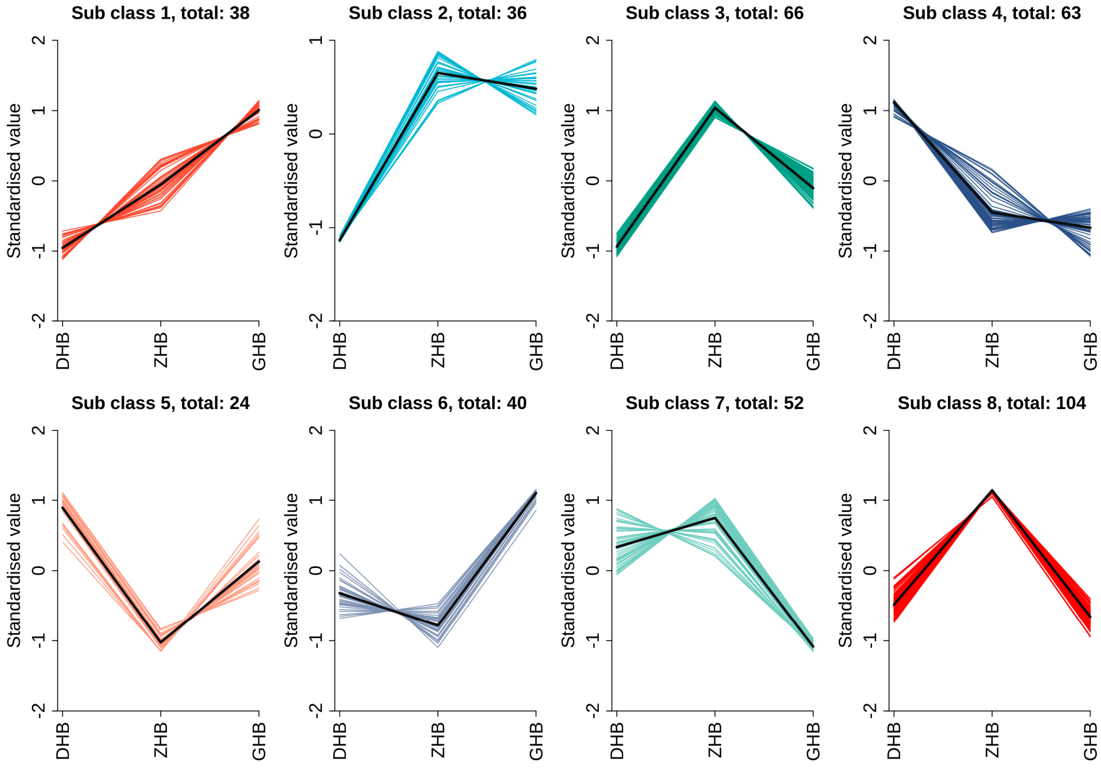

Figure S2. Trend-based clustering of differentially accumulated flavonoids across altitudinal groups.The 423 differentially accumulated flavonoids (DAFs) were grouped into eight accumulation subclasses (Sub class 1–8) showing Z-score standardised profiles across DHB, ZHB and GHB. Coloured lines represent individual flavonoids and the black line indicates the mean trend; “total” denotes the number of flavonoids in each subclass.The 423 DAFs were clustered into eight accumulation trend subclasses

Figure S3. Representative morphology of sampled *Chimonobambusa utilis* plants and leaves.

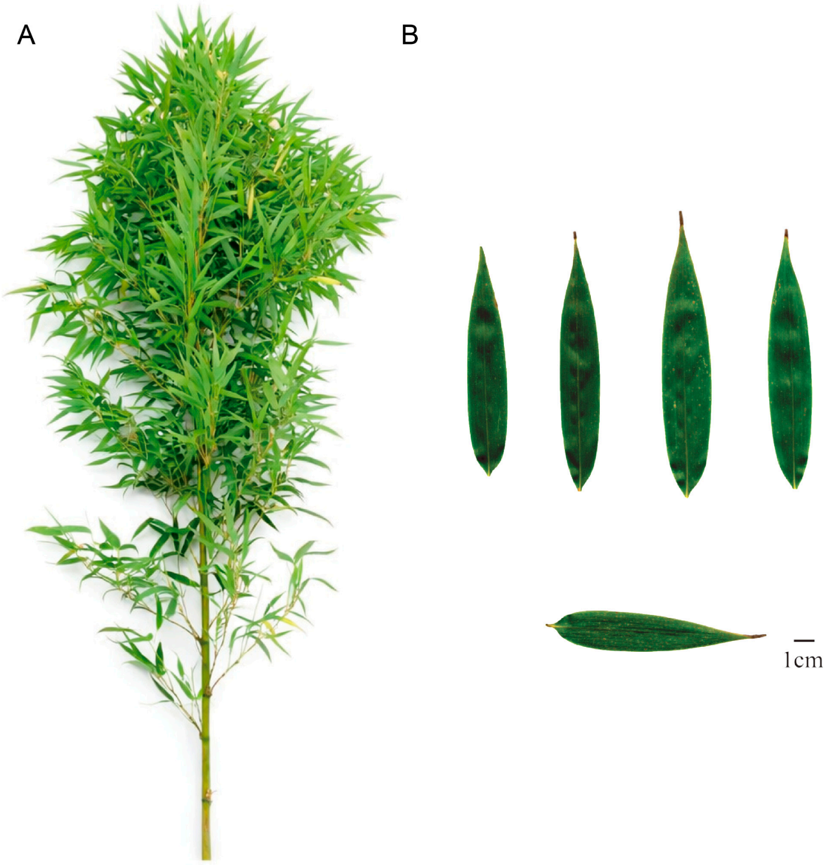

Figure S3. Representative morphology of sampled *Ch. utilis* plants and leaves. (A) Representative shoot used for sampling. (B) Representative leaves showing leaf shape and size.
