# Supplementary figures and images for: Elevational Metabolic Reprogramming Optimizes Flavonoid Accumulation and Antioxidant Capacity in Chimonobambusa utilis Leaves
Source: Plants (Basel). 2026 Apr 22;15(9):1290. doi: 10.3390/plants15091290 (PMC13165436; doi:10.3390/plants15091290)

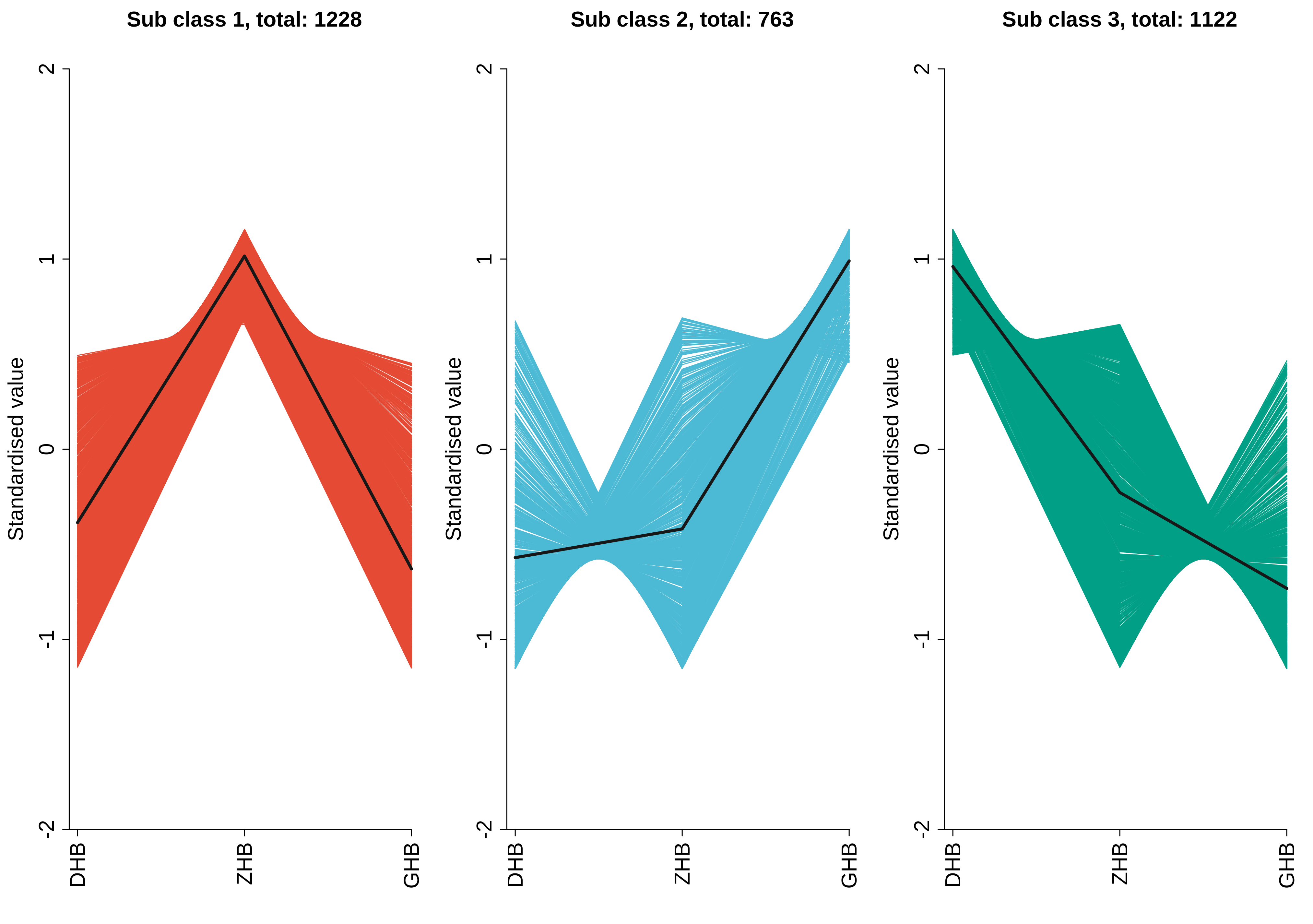

Supplement: Supplementary file 1 [file plants-15-01290-s001.zip › Supplementary Figure 1.png]

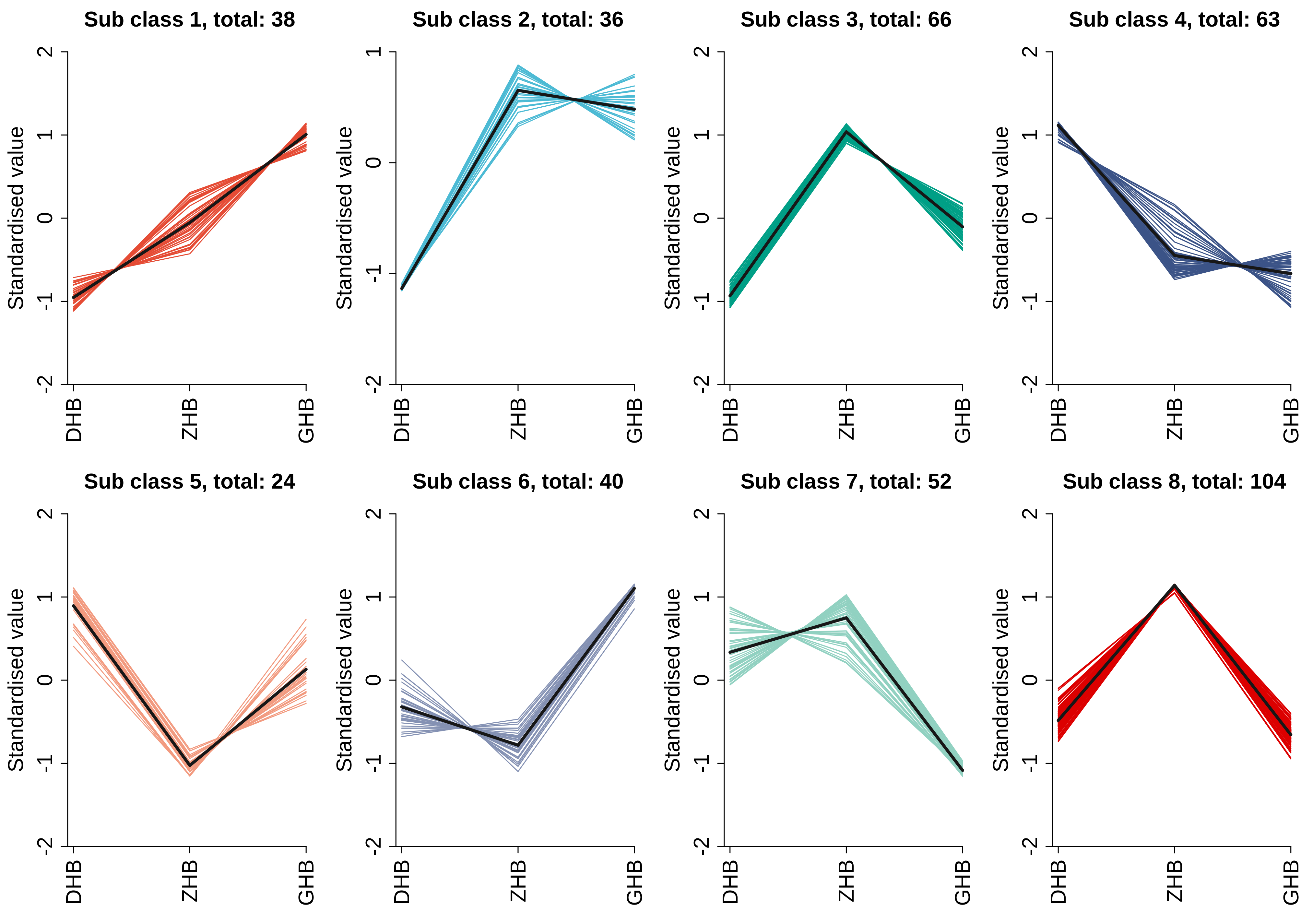

Supplement: Supplementary file 1 [file plants-15-01290-s001.zip › Supplementary Figure 2.png]

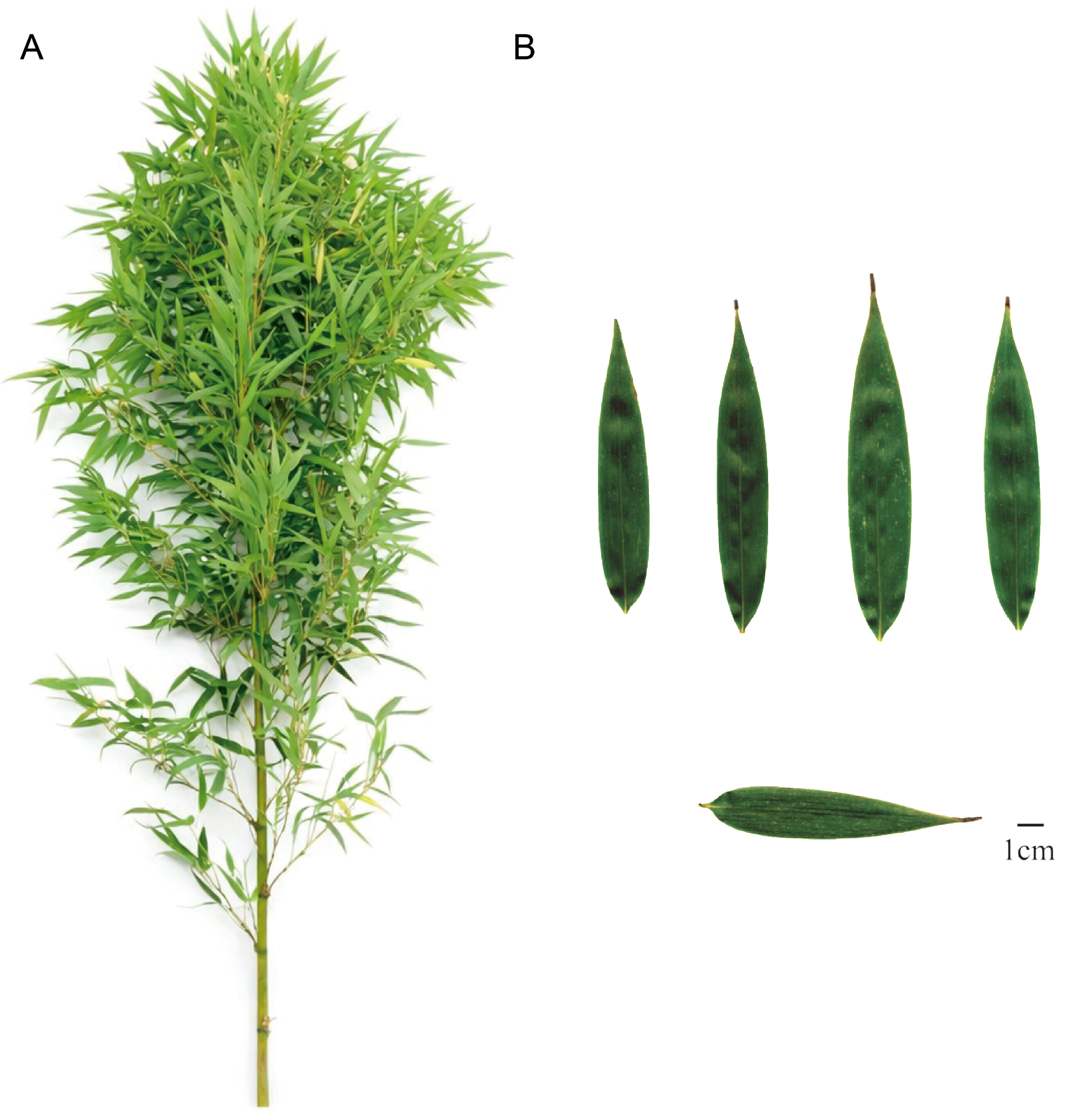

Supplement: Supplementary file 1 [file plants-15-01290-s001.zip › Supplementary Figure 3.png]
